# IL-13Rα2 Regulates C2C12 Myoblast Proliferation via the Akt–Cyclin D1–CDK4 Pathway

**DOI:** 10.3390/ijms27125600

**Published:** 2026-06-21

**Authors:** Mitsutoshi Kurosaka, Kazuhisa Kohda

**Affiliations:** Department of Physiology, St. Marianna University School of Medicine, 2-16-1 Sugao, Miyamae-ku, Kawasaki 216-8511, Kanagawa, Japan; kkohda@marianna-u.ac.jp

**Keywords:** IL-13Rα2, myoblast proliferation, Akt signaling, Cyclin D1, skeletal muscle, cytokine signaling

## Abstract

Interleukin-13 receptor α2 (IL-13Rα2) has traditionally been considered a decoy receptor; however, its cellular functions beyond the immune system remain unclear. We aimed to investigate the role of IL-13Rα2 in C2C12 myoblast proliferation and differentiation. IL-13Rα2 expression was knocked down in C2C12 cells using siRNA. Myogenic differentiation was evaluated by myosin heavy chain (MyHC) immunostaining and by quantifying the expression of myogenic regulatory and fusion-related genes. Myoblast proliferation was assessed using BrdU incorporation and cell number analyses, and signaling events induced by IL-13Rα2 knockdown were analyzed via immunoblotting and immunocytochemical analysis. IL-13Rα2 knockdown did not alter myogenic differentiation or the expression of fusion-associated genes. In contrast, IL-13Rα2 knockdown significantly increased BrdU incorporation and cell number, accompanied by increased Akt phosphorylation and decreased ERK phosphorylation. Cyclin D1 and cyclin-dependent kinase 4 (CDK4) levels were also increased. Akt inhibition abolished the enhanced proliferation and normalized Cyclin D1/CDK4 levels, whereas ERK activation did not further modify the knockdown-associated phenotype. These findings demonstrate that IL-13Rα2 negatively regulates myoblast proliferation by modulating the Akt–Cyclin D1–CDK4 signaling pathway, while being dispensable for myogenic differentiation.

## 1. Introduction

Skeletal muscle possesses a remarkable capacity for repair and regeneration throughout life. This regenerative ability is mediated primarily by satellite cells, which are tissue-resident muscle stem cells located beneath the basement membrane and adjacent to the sarcolemma of myofibers [1]. In their quiescent state, satellite cells contribute minimally to tissue turnover; however, upon activation, they differentiate into myoblasts that proliferate, undergo differentiation, and fuse with existing myofibers or with each other to form new fibers [1]. These processes maintain the myonuclear domain, restore muscle mass, and facilitate the repair of localized damage. A wide range of growth factors, cytokines, and extracellular cues orchestrate these transitions in myoblast states, highlighting the complexity of their regulatory networks [2].

Among cytokines implicated in myogenesis, interleukin-4 (IL-4) promotes myoblast fusion and contributes to myofiber growth and repair. IL-4 exerts its biological effects through IL-4 receptor α (IL-4Rα), which also participates in interleukin-13 (IL-13) signaling. Upon IL-13 binding to IL-13 receptor α1 (IL-13Rα1), IL-4Rα is recruited to form the type II IL-4/IL-13 receptor complex [3]. Given the well-documented role of IL-4 in promoting myoblast fusion and skeletal muscle growth [4], together with the shared receptor components between IL-4 and IL-13 signaling, IL-13 may also contribute to the regulation of myoblast behavior.

IL-13 receptor α2 (IL-13Rα2), in contrast, has been considered a decoy receptor that sequesters IL-13 and prevents IL-13Rα1-dependent signaling [5]. Consistent with this view, IL-13Rα2 has been shown to neutralize IL-13 activity in vivo and suppress IL-13-dependent inflammatory signaling [6,7]. However, accumulating evidence demonstrates that IL-13Rα2 can also function as a signaling receptor in a cell type-dependent manner. In fibroblasts, epithelial cells, macrophages, and various tumor cells, IL-13Rα2 has been reported to activate intracellular signaling pathways, including ERK1/2, AP-1, and related signaling pathways, thereby regulating cell proliferation, migration, and gene expression [8,9,10,11]. These findings challenge the traditional view of IL-13Rα2 as a purely inhibitory decoy receptor and suggest that it may exert active regulatory functions depending on cellular context.

Although both IL-13Rα1 and IL-13Rα2 are expressed in skeletal muscle cells [12], their specific roles in myoblast behavior are poorly understood. In particular, whether IL-13Rα2 contributes to the regulation of myoblast proliferation and differentiation, the two fundamental processes underlying skeletal muscle growth and regeneration, remains unclear. Therefore, this study aimed to investigate the functional role of IL-13Rα2 in C2C12 myoblasts and to identify the underlying signaling mechanisms. We demonstrate that IL-13Rα2 negatively regulates myoblast proliferation by modulating the Akt–Cyclin D1–CDK4 signaling pathway, while exerting little effect on the differentiation index, the number of myonuclei per myotube, and the expression of genes related to myogenic differentiation and fusion.

## 2. Results

### 2.1. IL-13Rα2 Knockdown Does Not Affect C2C12 Myoblast Differentiation

**Figure 1:**
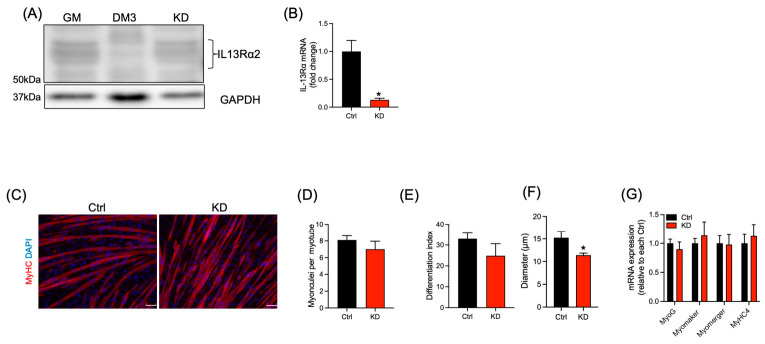
Effects of IL-13Rα2 knockdown on myogenic differentiation and myotube morphology in C2C12 cells. (**A**) Representative Western blot analysis of IL-13Rα2 protein expression. GM and DM3 indicate cells cultured in growth medium and differentiation medium for 3 days, respectively. KD indicates IL-13Rα2 siRNA-transfected myoblasts cultured in growth medium. The marked band corresponds to IL-13Rα2. (**B**) Relative IL-13Rα2 mRNA expression measured by quantitative PCR in control siRNA-transfected (Ctrl) and IL-13Rα2 siRNA-transfected (KD) cells. *n* = 4 per group. (**C**) Representative immunofluorescence images of Myosin Heavy Chain (MyHC; red) in differentiated myotubes. Nuclei were counterstained with DAPI (blue). Scale bars: 50 μm. (**D**) Quantification of the number of nuclei per myotube in the Ctrl and KD groups. *n* = 4 per group. (**E**) Differentiation index, calculated as the percentage of nuclei located within MyHC-positive myotubes. *n* = 4 per group. (**F**) Quantification of myotube diameter. *n* = 4 per group. (**G**) mRNA expression of myogenic differentiation- and fusion-related genes (MyoG, Myomaker, Myomerger, and MyHC4), as measured by qRT-PCR. *n* = 6 per group. Data are presented as mean ± SD. Statistical significance was determined using an unpaired Student’s *t*-test. * p < 0.05.

We first examined whether IL-13Rα2 influences myogenic differentiation, given that IL-4 signaling promotes myoblast differentiation and fusion. IL-13Rα2 expression was effectively reduced in C2C12 myoblasts by siRNA-mediated knockdown (Figure 1A,B). Differentiation was assessed by quantifying the number of myonuclei per myotube (Figure 1C,D) and the differentiation index (Figure 1C,E). Neither the number of myonuclei per myotube (*n* = 4, *p* = 0.15) nor the differentiation index (*n* = 4, *p* = 0.09) differed significantly between IL-13Rα2-KD and Ctrl cells. A significant reduction in myotube diameter was observed (Figure 1F, *n* = 4, *p* = 0.01).

To further evaluate the role of IL-13Rα2 in myoblast differentiation, we analyzed mRNA levels of key regulators of myogenic differentiation and myoblast fusion, including MyoG, Myomaker, Myomerger, and MyHC4. qRT-PCR analysis revealed no significant differences in the expression of MyoG (*p* = 0.19), Myomaker (*p* = 0.34), Myomerger (*p* = 0.70), or MyHC4 (*p* = 0.25) between IL-13Rα2-KD and Ctrl cells (Figure 1G; *n* = 6). These findings indicate that IL-13Rα2 knockdown does not substantially affect the differentiation index, myonuclei number per myotube, or the expression of myogenic differentiation- and fusion-related genes.

### 2.2. IL-13Rα2 Knockdown Promotes C2C12 Myoblast Proliferation

**Figure 2:**
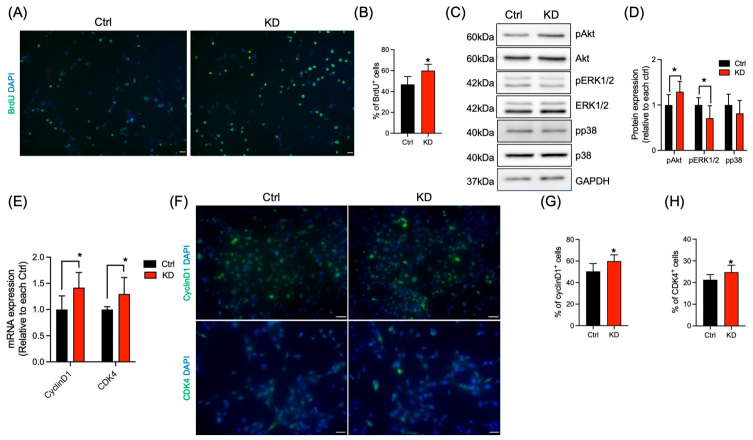
IL-13Rα2 knockdown enhances proliferation and alters Akt and ERK phosphorylation in C2C12 myoblasts. (**A**) Representative BrdU immunofluorescence images of Ctrl and KD cells cultured in the growth medium (GM). Nuclei were stained with DAPI (blue). Scale bars: 50 μm. (**B**) Quantification of BrdU-positive cells (green) showing significantly increased proliferation in KD cells. *n* = 6 per group. (**C**) Representative Western blots showing total and phosphorylated forms of Akt, ERK1/2, and p38. GAPDH was used as the loading control. (**D**) Quantification of pAkt, pERK1/2, and pp38 levels, as the ratio to their respective total protein levels. *n* = 6 per group. (**E**) qRT-PCR analysis of Cyclin D1 and CDK4 mRNA expression. *n* = 6 per group. (**F**–**H**) Representative immunofluorescence images and quantification of Cyclin D1-positive and CDK4-positive cells (green) in the Ctrl and KD groups. *n* = 6 per group. Scale bars: 50 μm. Data are presented as mean ± SD. Statistical significance was determined using an unpaired Student’s *t*-test. * *p* < 0.05.

We next examined whether IL-13Rα2 regulates myoblast proliferation. Under GM conditions, BrdU incorporation was significantly higher in the IL-13Rα2-KD group than in the Ctrl group, indicating enhanced cell-cycle entry (Figure 2A, B; *n* = 6, *p* = 0.01). Consistent with the BrdU incorporation results, the total cell number within the same microscopic fields was also significantly increased in IL-13Rα2-KD cells (Figure S2A; *n* = 4, *p* = 0.04).

To identify the signaling pathways involved, we examined the activation of Akt, ERK, and p38. As shown in Figure 2C,D, Western blot analysis revealed that IL-13Rα2-KD markedly increased phosphorylated Akt (pAkt) and decreased phosphorylated ERK (pERK), but did not alter phosphorylated p38 (pp38) (Figure 2C,D; *n* = 6; *p* = 0.04 for pAkt, *p* = 0.04 for pERK, and *p* = 0.21 for pp38).

Because Akt and ERK signaling can regulate cell-cycle entry through Cyclin D1 and CDK4 [13,14], we then analyzed downstream cell-cycle regulators and found that IL-13Rα2-KD significantly increased Cyclin D1 and CDK4 mRNA levels (Figure 2E; *n* = 6; *p* = 0.02 for Cyclin D1, *p* = 0.04 for CDK4). Immunostaining revealed a significantly higher proportion of Cyclin D1- and CDK4-positive cells in the KD group (Figure 2F–H; *n* = 6; *p* = 0.03 for Cyclin D1, *p* = 0.04 for CDK4). These results suggest that IL-13Rα2 limits myoblast proliferation via Akt and/or ERK signaling, which in turn downregulates Cyclin D1 and CDK4.

### 2.3. Akt Inhibition Abolishes the Pro-Proliferative Effect of IL-13Rα2 Knockdown

**Figure 3:**
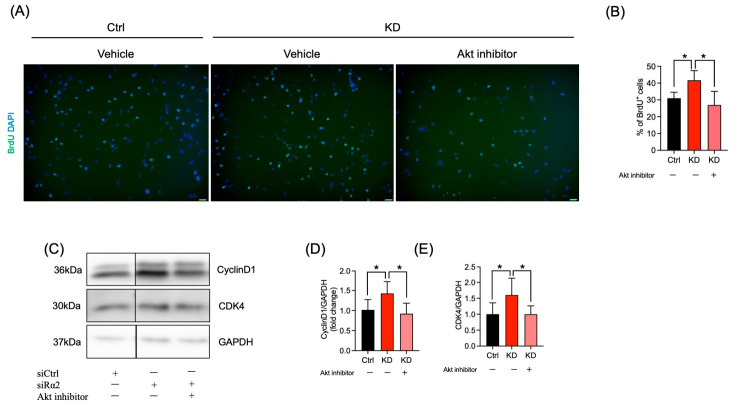
Akt inhibition abolishes the enhanced proliferation and Cyclin D1/CDK4 upregulation caused by IL-13Rα2 knockdown. (**A**) Representative BrdU staining of Ctrl, KD, and KD cells treated with an Akt inhibitor (10 μM MK-2206). Scale bar = 50 μm. (**B**) Quantification of BrdU-positive cells (green). The promotion of proliferation due to KD was reversed by Akt inhibition. *n* = 6 per group. (**C**) Representative Western blots of Cyclin D1 and CDK4 in Ctrl, KD, and KD + Akt inhibitor groups. (**D**,**E**) Quantification of Cyclin D1 and CDK4 protein levels, showing that Akt inhibition suppresses KD-induced upregulation. *n* = 6 per group. Data are presented as mean ± SD. Statistical analysis was performed using one-way ANOVA with Tukey–Kramer *post hoc* test for multiple comparisons. * *p* < 0.05.

To determine whether Akt activation is required for the increased proliferation induced by IL-13Rα2-KD, we employed the allosteric Akt inhibitor MK-2206. In IL-13Rα2-KD myoblasts, treatment with 10 μM MK-2206 restored both the number of BrdU-positive cells (Figure 3B; *n* = 6, *p* = 0.04 for Ctrl vs. KD and *p* = 0.006 for KD vs. KD + MK-2206) and the total cell count (Figure S2B; *n* = 6, *p* = 0.02 for Ctrl vs. KD and *p* = 0.001 for KD vs. KD + MK-2206) to control levels, thereby reversing the KD-induced increase in proliferation.

We next assessed downstream regulators of G1–S progression. Cyclin D1 and CDK4 were elevated in IL-13Rα2-KD cells but were restored to the Ctrl level when Akt was inhibited (Figure 3C–E; *n* = 6; *p* = 0.04 and *p* = 0.04 for Ctrl vs. KD, and *p* = 0.01 and *p* = 0.04 for KD vs. KD + MK-2206, respectively). Together, these results demonstrate that loss of IL-13Rα2 leads to Akt-dependent activation of the cell-cycle regulators Cyclin D1 and CDK4, which in turn promotes DNA synthesis and proliferative activity. These findings suggest that the Akt–Cyclin D1–CDK4 signaling pathway contributes, at least in part, to the enhanced proliferation observed following IL-13Rα2 knockdown.

### 2.4. ERK Activation Does Not Rescue the IL-13Rα2 Knockdown Phenotype

**Figure 4:**
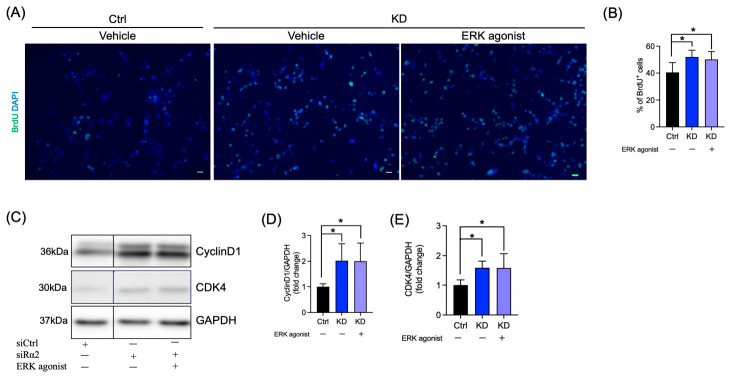
ERK activation does not modify the proliferative phenotype of IL-13Rα2 knockdown myoblasts. (**A**) Representative BrdU staining of Ctrl, KD, and KD cells treated with the ERK activator (rel)-AR234960 (10 μM). Scale bar = 50 μm. (**B**) Quantification of BrdU-positive cell (green) numbers showing no significant difference between KD and KD + ERK activator groups. *n* = 6 per group. (**C**) Representative Western blots of Cyclin D1 and CDK4. (**D**,**E**) Quantification showing elevated Cyclin D1/CDK4 protein levels in KD cells relative to Ctrl, with no further increase after ERK activation. *n* = 5 per group. Data are presented as mean ± SD. Statistical analysis was performed using one-way ANOVA with Tukey–Kramer *post hoc* test for multiple comparisons. * *p* < 0.05.

Although activation of ERK signaling canonically promotes cell proliferation in various cell types, the enhanced proliferation of IL-13Rα2-KD C2C12 cells was associated with reduced ERK phosphorylation (Figure 2). We investigated whether ERK signaling is involved in the proliferation of IL-13Rα2-KD C2C12 cells using the ERK agonist (rel)-AR234960. Treatment with 10 μM (rel)-AR234960 did not affect the increased BrdU incorporation (Figure 4A,B; *n* = 6, *p* = 0.86 for KD vs. KD + (rel)-AR234960) or the total cell number in IL-13Rα2-KD cells (Figure S2C; *n* = 6; *p* = 0.64 for KD vs. KD + (rel)-AR234960). Furthermore, the elevated expression levels of Cyclin D1 and CDK4 in IL-13Rα2-KD cells were not reversed by ERK agonist treatment (Figure 4C–E; *n* = 5; *p =* 0.99 for Cyclin D1 and *p* = 0.99 for CDK4 in KD vs. KD + (rel)-AR234960). Thus, pharmacological activation of ERK was insufficient to reverse the proliferative phenotype induced by IL-13Rα2 knockdown under the present experimental conditions.

## 3. Discussion

In this study, we investigated the functional role of IL-13Rα2 in C2C12 myoblasts. We found that IL-13Rα2 knockdown significantly promoted myoblast proliferation without affecting the differentiation index, the number of nuclei per myotube, or the expression of myogenic differentiation- and fusion-related genes. Mechanistically, loss of IL-13Rα2 resulted in increased Akt phosphorylation and upregulation of Cyclin D1 and CDK4 expression. Furthermore, the Akt inhibitor MK-2206 abolished the pro-proliferative effects of IL-13Rα2 knockdown and reversed the activation of Akt–Cyclin D1–CDK4 signaling. These findings suggest that IL-13Rα2 functions as a negative regulator of myoblast proliferation, at least in part, through modulation of Akt-mediated cell-cycle progression.

While IL-13Rα2 has been considered a decoy receptor that sequesters IL-13 to antagonize IL-13Rα1-dependent signaling [5], emerging evidence suggests that IL-13Rα2 can also function as a signaling receptor in several cell types [9,10,11,15]. Our findings that IL-13Rα2 knockdown significantly promoted cell proliferation and activated signaling pathways that regulate cell-cycle progression strongly support the idea that IL-13Rα2 functions as an active signal transducer in C2C12 cells. One could argue that these pro-proliferative effects are driven by unopposed IL-13Rα1 activity following the loss of the IL-13Rα2 decoy effect. However, this is unlikely because the type II IL-4/IL-13 receptor complex (IL-4Rα/IL-13Rα1) is known to promote myoblast differentiation and fusion rather than proliferation [16]. Consequently, the functional balance between IL-13Rα2 and the type II IL-4/IL-13 receptor complex should be a critical determinant of myoblast fate.

We demonstrated that the enhancement of myoblast proliferation via IL-13Rα2 knockdown was accompanied by Akt phosphorylation and ERK dephosphorylation. This is seemingly paradoxical, since both PI3K/Akt and ERK pathways are traditionally implicated in the positive regulation of cell-cycle progression [17,18,19]. However, our pharmacological experiments clarified the distinct functional contributions of these pathways. Inhibition of Akt completely abolished the enhanced BrdU incorporation and normalized the expression levels of Cyclin D1 and CDK4 in IL-13Rα2 knockdown cells, indicating that Akt activation is indispensable for the pro-proliferative phenotype. Consistent with our results, previous studies in various cell types indicated that PI3K/Akt signaling pathways drive the G1–S transition by modulating Cyclin D1 and CDK activity [17,18]. Furthermore, it has been reported that PI3K/Akt signaling suppresses Cyclin D1 turnover by inhibiting GSK-3β–mediated phosphorylation [20] and prevents the nuclear translocation of p27, thereby promoting cell-cycle progression [21]. Collectively, our data suggest that Akt signaling serves as a pivotal mediator of the myoblast cell cycle progression by orchestrating the abundance, activity, and subcellular localization of key regulatory molecules.

By contrast, pharmacological activation of ERK failed to reverse the pro-proliferative effects of IL-13Rα2 knockdown, nor did it further augment proliferation or the expression of Cyclin D1 and CDK4. Given the well-documented crosstalk and reciprocal negative feedback between the PI3K/Akt and RAF–MEK–ERK pathways [22,23], Akt activation induced by IL-13Rα2 knockdown may suppress ERK signaling in C2C12 cells. Therefore, the inability of ERK activation to reverse the pro-proliferative phenotype may reflect the predominance of Akt-mediated signaling under our experimental conditions. In addition, we cannot exclude the possibility that the efficacy, concentration, or duration of ERK agonist treatment was insufficient to reveal a contribution of ERK signaling to the regulation of myoblast proliferation.

This study has several limitations. First, proliferative activity was assessed by BrdU incorporation together with total cell number quantification, both of which suggested enhanced proliferative activity following IL-13Rα2 knockdown. However, these measurements primarily reflect DNA synthesis and changes in cell number and cannot fully exclude the potential contribution of other factors, including altered cell survival, cell death, or cell-cycle arrest. Additional analyses, such as growth-curve assays, apoptosis assays, and comprehensive cell-cycle profiling, will be necessary to more definitively evaluate the effects of IL-13Rα2 on myoblast proliferation. Second, myotube differentiation was assessed only at DM3, and a time-course analysis at later differentiation stages was not performed in this study. Therefore, we cannot exclude the possibility that IL-13Rα2 knockdown delays myogenic differentiation or affects later stages of myotube formation. In addition, IL-13Rα2 knockdown significantly reduced myotube diameter, suggesting that IL-13Rα2 may contribute to certain aspects of myotube growth or maturation. Future studies including later differentiation time points and protein-level analyses are warranted to determine whether IL-13Rα2 influences the progression of myogenic differentiation or later stages of myotube maturation. Third, the specific molecular intermediates linking IL-13Rα2 to regulation of Akt signaling remain to be elucidated. Previous studies have suggested that the cytoplasmic domain of IL-13Rα2 is associated with intracellular signaling pathways, including AP-1-related and EGFR-associated pathways [8,15,24]. Further investigation is required to define these downstream mechanisms. Another limitation of this study is that the involvement of IL-13 signaling was not directly examined. Because all experiments were conducted under basal culture conditions without exogenous IL-13 stimulation, it remains unclear whether the observed effects of IL-13Rα2 knockdown reflect ligand-dependent IL-13 signaling or ligand-independent functions of IL-13Rα2. Further studies using IL-13 stimulation under IL-13Rα2-deficient conditions will be required to fully clarify the contribution of IL-13 signaling to the regulation of myoblast proliferation. Finally, as this study was conducted using the C2C12 cell line, the functional role of IL-13Rα2 should be validated in more physiologically relevant systems. Notably, in vivo, IL-13 is primarily produced by infiltrating immune cells within regenerating muscle rather than by myoblasts or myofibers themselves [25,26]. This highlights the potential importance of the signaling balance between IL-13Rα1 and IL-13Rα2 in regulating myogenic progenitor cell dynamics. A more comprehensive understanding of IL-13 signaling will require further studies to elucidate how these receptor-mediated pathways are integrated.

## 4. Materials & Methods

### 4.1. Cell Culture

C2C12 myoblasts (American Type Culture Collection [ATCC], Manassas, VA, USA) were maintained in a growth medium (GM) comprising Dulbecco’s modified Eagle’s medium (DMEM; Life Technologies Inc., Carlsbad, CA, USA) supplemented with 10% bovine growth serum (Cytiva, Tokyo, Japan) and 1% penicillin–streptomycin (Life Technologies Inc., Carlsbad, CA, USA). To induce myogenic differentiation, GM was replaced with differentiation medium (DM), comprising DMEM supplemented with 2% horse serum (Life Technologies Inc.) and 1% penicillin–streptomycin.

### 4.2. BrdU Incorporation Assay

C2C12 cells were cultured in GM medium for 24 h, with the final 2 h spent incubating with 10 μM 5-bromo-2′-deoxyuridine (BrdU). BrdU incorporation was detected by immunocytochemistry as previously described [27]. The number of BrdU-positive nuclei was measured in four randomly selected fields of view per sample. The mean value of these counts was used as the BrdU-positive cell count for each sample in subsequent statistical analyses. Total cell number was also quantified by counting DAPI-positive nuclei in the same microscopic fields used for BrdU analysis. The images were acquired using a fluorescence microscope (BZ-9000; Keyence Co., Ltd., Osaka, Japan).

### 4.3. siRNA Transfection

IL-13Rα2–specific Dicer-substrate siRNA (siIL-13Rα2) and control siRNA (siCtrl) were purchased from Integrated DNA Technologies (IDT; San Diego, CA, USA). Transfections were performed using Lipofectamine RNAiMAX (Life Technologies Inc.) according to the manufacturer’s protocol. After transfection, cells were cultured in GM for 24 h before being switched to fresh GM or DM for proliferation and differentiation assays, respectively.

### 4.4. Quantitative Real-Time PCR (qRT-PCR)

Total RNA was isolated with Sepasol-RNA I Super G (Nacalai Tesque Inc., Kyoto, Japan) and purified using RNA purification columns (Favorgen Biotech Corp., Ping-Tung, Taiwan), as previously described [27]. RNA concentration and purity were assessed spectrophotometrically prior to downstream analyses. First-strand cDNA was synthesized from 1 μg of total RNA using ReverTra Ace qPCR Master Mix (Toyobo Co., Ltd., Osaka, Japan). Quantitative real-time PCR (qRT-PCR) was performed using a StepOnePlus Real-Time PCR System (Life Technologies Inc., Carlsbad, CA, USA) with SYBR Green Master Mix (Toyobo Co., Ltd.). The PCR cycling conditions were 95 °C for 1 min, followed by 40 cycles of 95 °C for 15 s and 60 °C for 30 s. Relative gene expression levels were calculated using the 2−ΔΔCt method, with GAPDH as the internal reference gene. The primer sequences used in this study are listed in Supplementary Table S1.

### 4.5. Immunocytochemistry

Immunocytochemical staining was performed as previously described [16,27]. Briefly, C2C12 cells were washed with phosphate-buffered saline (PBS) and fixed in ice-cold methanol for 15 min. After blocking with 3% bovine serum albumin (BSA) in PBS for 30 min, cells were incubated at 4 °C overnight with primary antibodies against myosin heavy chain (MyHC, 1:50; DSHB, Iowa City, IA, USA), Cyclin D1 (1:200; Santa Cruz Biotechnology Inc., Dallas, TX, USA), and cyclin-dependent kinase 4 (CDK4, 1:50; Proteintech, Rosemont, IL, USA). Cells were then incubated with Alexa Fluor 488- or 546-conjugated secondary antibodies (Life Technologies Inc.) for 1 h at room temperature. Nuclei were counterstained with DAPI (Sigma-Aldrich, St. Louis, MO, USA). Images were obtained using a BZ-9000 microscope (Keyence Co., Ltd., Osaka, Japan) and analyzed with Fiji software, version 2.14.0 (National Institutes of Health, Bethesda, MD, USA). Differentiation index and the number of nuclei per myotube were calculated from four randomly selected fields of view per sample. The differentiation index was defined as the percentage of nuclei located within MyHC-positive multinucleated myotubes relative to the total number of nuclei. The number of myonuclei per myotube was calculated by dividing the total number of nuclei within MyHC-positive myotubes by the total number of myotubes. Myotube diameter was measured using Fiji software. For each sample, four microscopic visual fields were randomly selected, and 10 myotubes were measured per field. The diameter of each myotube was determined as the mean of measurements obtained at three distinct locations along its length. These data were averaged to serve as the representative value for each independent experiment.

### 4.6. Western Blotting

Cells were lysed in RIPA buffer (Wako, Osaka, Japan) supplemented with protease and phosphatase inhibitors (Nacalai Tesque Inc.). Protein concentrations were determined using a BCA protein assay kit (Thermo Fisher Scientific, Waltham, MA, USA). Equal amounts of protein (20 μg) were separated by SDS–PAGE at 120 V for 60 min and transferred onto PVDF membranes (Cytiva, Marlborough, MA, USA) by wet transfer at 100 V for 60 min. Western blotting was performed as previously described [16,27]. Briefly, the membranes were blocked with Blocking buffer (Nacalai Tesque Inc.) for 1 h at room temperature and incubated overnight at 4 °C with the following primary antibodies: anti-IL-13Rα2 (1:1000; Proteintech, Rosemont, IL, USA), anti-phospho-Akt (Ser473, 1:2000; Cell Signaling Technology [CST], Danvers, MA, USA), anti-Akt (1:2000; CST), anti-phospho-ERK1/2 (Thr202/Tyr204, 1:2000; CST), anti-ERK1/2 (1:2000; CST), anti-phospho-p38 MAPK (Thr180/Tyr182, 1:1000; CST), anti-p38 (1:1000; CST), and anti-GAPDH (1:2000; CST). The membranes were then incubated with HRP-conjugated secondary antibodies (1:5000; CST) for 1 h at room temperature. Signals were detected using enhanced chemiluminescence (ECL) reagents (Bio-Rad Laboratories Inc., Hercules, CA, USA) and imaged using a LAS-4000 imaging system (Fujifilm Corp., Tokyo, Japan). Densitometric analysis was performed using Fiji software. Phosphorylated Akt, ERK1/2, and p38 were normalized to their respective total proteins, whereas Cyclin D1 and CDK4 were normalized to GAPDH.

### 4.7. Pharmacological Modulation of Akt and ERK

For pharmacological experiments, C2C12 myoblasts were transfected with control or IL-13Rα2 siRNA. Twenty-four hours after transfection, cells were treated with the Akt inhibitor MK-2206 (10 μM; MedChemExpress, Monmouth Junction, NJ, USA) or the selective ERK activator (rel)-AR234960 (10 μM; MedChemExpress) for 24 h in growth medium (GM). Vehicle control cells received an equivalent volume of DMSO (Sigma-Aldrich, St. Louis, MO, USA). Following treatment, cells were processed for BrdU incorporation assays or harvested for Western blot analysis.

### 4.8. Statistical Analysis

All data are presented as mean ± standard deviation (SD). Statistical analyses were performed using Prism v10.0 (GraphPad Software Inc., San Diego, CA, USA). Comparisons between two groups were made using unpaired Student’s *t*-tests, and multiple-group comparisons were evaluated using one-way ANOVA followed by Tukey–Kramer *post hoc* tests. *p*-values less than 0.05 were considered statistically significant.

## 5. Conclusions

Our study demonstrates that IL-13Rα2 acts as a negative regulator of myoblast proliferation, at least in part, by suppressing the Akt–Cyclin D1–CDK4 signaling pathway. Given that the type II IL-4/IL-13 receptor complex (IL-4Rα/IL-13Rα1) is involved in myogenic differentiation, the functional balance between IL-13Rα1 and IL-13Rα2 emerges as a critical determinant of myoblast fate. Further investigations are warranted to elucidate the precise molecular mechanisms underlying IL-13Rα2-mediated signaling in skeletal muscle lineage cells.

## Data Availability

All relevant data are contained within the manuscript and its Supplementary Materials.

## Supplementary Materials

The following supporting information can be downloaded at: https://www.mdpi.com/article/10.3390/ijms27125600/s1.

## Abbreviations

IL-4: interleukin-4; IL-4Rα, interleukin-4 receptor α; IL-13, interleukin-13; IL-13Rα1, interleukin-13 receptor α1; IL-13Rα2, interleukin-13 receptor α2; MyHC, myosin heavy chain; BrdU, 5-bromo-2′-deoxyuridine; CDK4, cyclin-dependent kinase 4; qRT-PCR, quantitative real-time polymerase chain reaction.

## References

[1] Chang N.C., Rudnicki M.A. (2014). Satellite cells: The architects of skeletal muscle. Curr. Top. Dev. Biol..

[2] Costamagna D., Costelli P., Sampaolesi M., Penna F. (2015). Role of Inflammation in Muscle Homeostasis and Myogenesis. Mediat. Inflamm..

[3] Wynn T.A. (2003). IL-13 effector functions. Annu. Rev. Immunol..

[4] Horsley V., Jansen K.M., Mills S.T., Pavlath G.K. (2003). IL-4 acts as a myoblast recruitment factor during mammalian muscle growth. Cell.

[5] Rahaman S.O., Sharma P., Harbor P.C., Aman M.J., Vogelbaum M.A., Haque S.J. (2002). IL-13R(alpha)2, a decoy receptor for IL-13 acts as an inhibitor of IL-4-dependent signal transduction in glioblastoma cells. Cancer Res..

[6] Chen W., Sivaprasad U., Tabata Y., Gibson A.M., Stier M.T., Finkelman F.D., Hershey G.K. (2009). IL-13R alpha 2 membrane and soluble isoforms differ in humans and mice. J. Immunol..

[7] Wilson M.S., Elnekave E., Mentink-Kane M.M., Hodges M.G., Pesce J.T., Ramalingam T.R., Thompson R.W., Kamanaka M., Flavell R.A., Keane-Myers A. (2007). IL-13Ralpha2 and IL-10 coordinately suppress airway inflammation, airway-hyperreactivity, and fibrosis in mice. J. Clin. Investig..

[8] Fichtner-Feigl S., Young C.A., Kitani A., Geissler E.K., Schlitt H.J., Strober W. (2008). IL-13 signaling via IL-13R alpha2 induces major downstream fibrogenic factors mediating fibrosis in chronic TNBS colitis. Gastroenterology.

[9] Yang S.J., Allahverdian S., Saunders A.D.R., Liu E., Dorscheid D.R. (2019). IL-13 signaling through IL-13 receptor alpha2 mediates airway epithelial wound repair. FASEB J..

[10] Jannoo R., Xia Z., Row P.E., Kanamarlapudi V. (2023). Targeting of the Interleukin-13 Receptor (IL-13R)alpha2 Expressing Prostate Cancer by a Novel Hybrid Lytic Peptide. Biomolecules.

[11] Knudson K.M., Hwang S., McCann M.S., Joshi B.H., Husain S.R., Puri R.K. (2022). Recent Advances in IL-13Ralpha2-Directed Cancer Immunotherapy. Front. Immunol..

[12] Prokopchuk O., Liu Y., Wang L., Wirth K., Schmidtbleicher D., Steinacker J.M. (2007). Skeletal muscle IL-4, IL-4Ralpha, IL-13 and IL-13Ralpha1 expression and response to strength training. Exerc. Immunol. Rev..

[13] Shimura T., Kakuda S., Ochiai Y., Kuwahara Y., Takai Y., Fukumoto M. (2011). Targeting the AKT/GSK3beta/cyclin D1/Cdk4 survival signaling pathway for eradication of tumor radioresistance acquired by fractionated radiotherapy. Int. J. Radiat. Oncol. Biol. Phys..

[14] Jia X., Liu B., Shi X., Ye M., Zhang F., Liu H. (2011). Roles of the ERK, JNK/AP-1/cyclin D1-CDK4 pathway in silica-induced cell cycle changes in human embryo lung fibroblast cells. Cell Biol. Int..

[15] Fichtner-Feigl S., Strober W., Kawakami K., Puri R.K., Kitani A. (2006). IL-13 signaling through the IL-13alpha2 receptor is involved in induction of TGF-beta1 production and fibrosis. Nat. Med..

[16] Kurosaka M., Hung Y.L., Machida S., Kohda K. (2023). IL-4 Signaling Promotes Myoblast Differentiation and Fusion by Enhancing the Expression of MyoD, Myogenin, and Myomerger. Cells.

[17] Fatrai S., Elghazi L., Balcazar N., Cras-Meneur C., Krits I., Kiyokawa H., Bernal-Mizrachi E. (2006). Akt induces beta-cell proliferation by regulating cyclin D1, cyclin D2, and p21 levels and cyclin-dependent kinase-4 activity. Diabetes.

[18] Li Y., Dowbenko D., Lasky L.A. (2002). AKT/PKB phosphorylation of p21Cip/WAF1 enhances protein stability of p21Cip/WAF1 and promotes cell survival. J. Biol. Chem..

[19] Meloche S., Pouyssegur J. (2007). The ERK1/2 mitogen-activated protein kinase pathway as a master regulator of the G1- to S-phase transition. Oncogene.

[20] Diehl J.A., Cheng M., Roussel M.F., Sherr C.J. (1998). Glycogen synthase kinase-3beta regulates cyclin D1 proteolysis and subcellular localization. Genes Dev..

[21] Liang J., Zubovitz J., Petrocelli T., Kotchetkov R., Connor M.K., Han K., Lee J.H., Ciarallo S., Catzavelos C., Beniston R. (2002). PKB/Akt phosphorylates p27, impairs nuclear import of p27 and opposes p27-mediated G1 arrest. Nat. Med..

[22] Mendoza M.C., Er E.E., Blenis J. (2011). The Ras-ERK and PI3K-mTOR pathways: Cross-talk and compensation. Trends Biochem. Sci..

[23] Aksamitiene E., Kiyatkin A., Kholodenko B.N. (2012). Cross-talk between mitogenic Ras/MAPK and survival PI3K/Akt pathways: A fine balance. Biochem. Soc. Trans..

[24] Newman J.P., Wang G.Y., Arima K., Guan S.P., Waters M.R., Cavenee W.K., Pan E., Aliwarga E., Chong S.T., Kok C.Y.L. (2017). Interleukin-13 receptor alpha 2 cooperates with EGFRvIII signaling to promote glioblastoma multiforme. Nat. Commun..

[25] Heredia J.E., Mukundan L., Chen F.M., Mueller A.A., Deo R.C., Locksley R.M., Rando T.A., Chawla A. (2013). Type 2 innate signals stimulate fibro/adipogenic progenitors to facilitate muscle regeneration. Cell.

[26] Burzyn D., Kuswanto W., Kolodin D., Shadrach J.L., Cerletti M., Jang Y., Sefik E., Tan T.G., Wagers A.J., Benoist C. (2013). A special population of regulatory T cells potentiates muscle repair. Cell.

[27] Kurosaka M., Ogura Y., Sato S., Kohda K., Funabashi T. (2021). Transcription factor signal transducer and activator of transcription 6 (STAT6) is an inhibitory factor for adult myogenesis. Skelet. Muscle.

