# Supplementary material for: IL-13Rα2 Regulates C2C12 Myoblast Proliferation via the Akt–Cyclin D1–CDK4 Pathway
Source: Int J Mol Sci. 2026 Jun 21;27(12):5600. doi: 10.3390/ijms27125600 (PMC13299564; doi:10.3390/ijms27125600)
Supplement: Supplementary file 1 [file ijms-27-05600-s001.zip › ijms-4323502-supplementary.pdf]

Figure S1. Uncropped Western Blot Images

Bands for Fig.1A

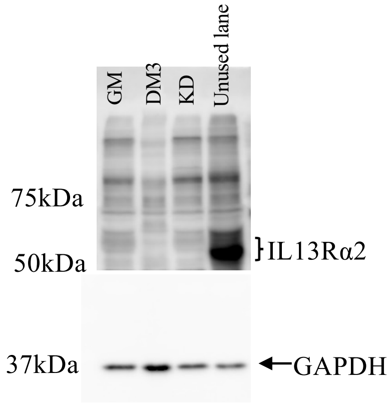

Figure S1. Uncropped Western Blot Images

Bands for Fig.2C

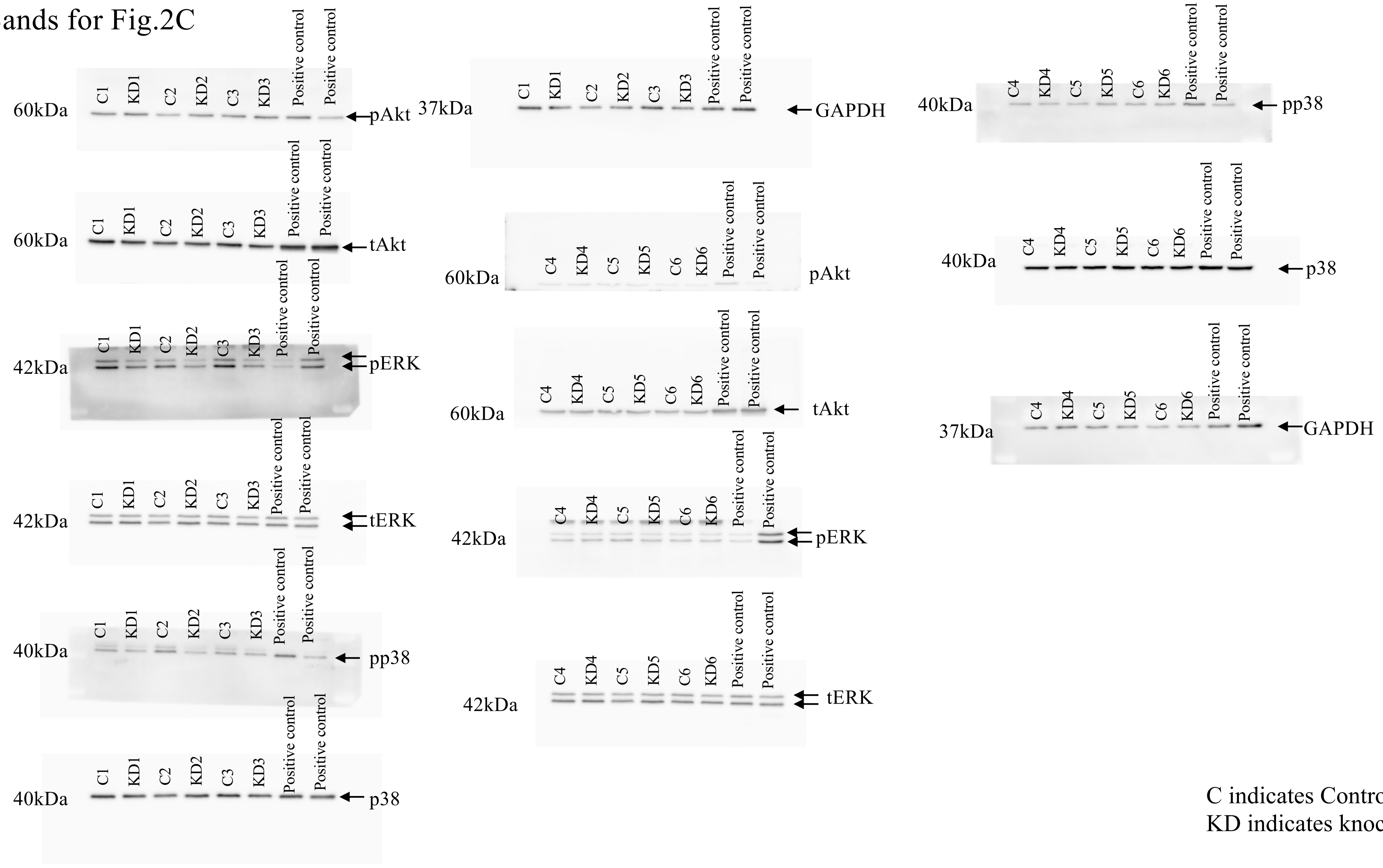

C indicates Control group.
KD indicates knockdown group.

Figure S1. Uncropped Western Blot Images

Bands for Fig.3C

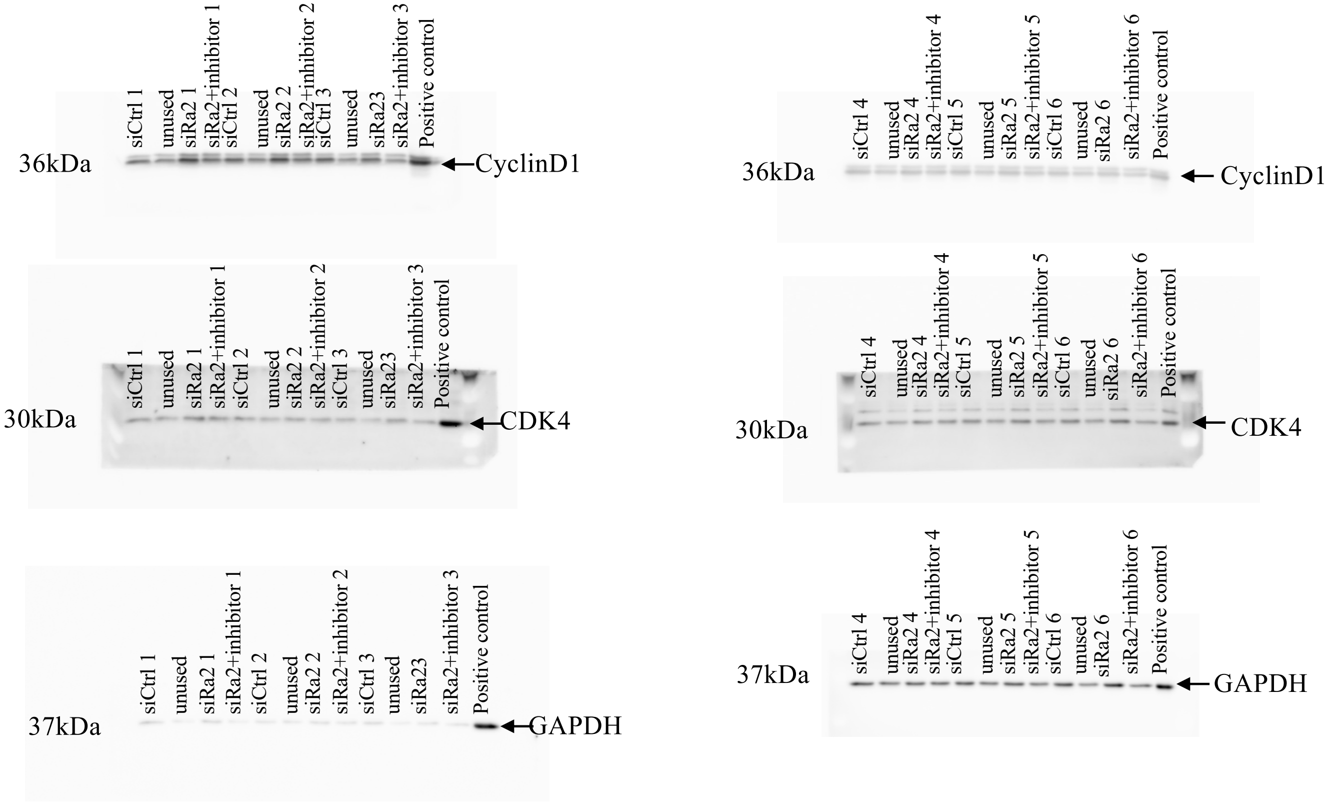

siCtrl indicates Control group.
siRa2 indicates knockdown group.
siRa2+inhibitor indicates knockdown cells treated with inhibitor.

Figure S1. Uncropped Western Blot Images

Bands for Fig.4C

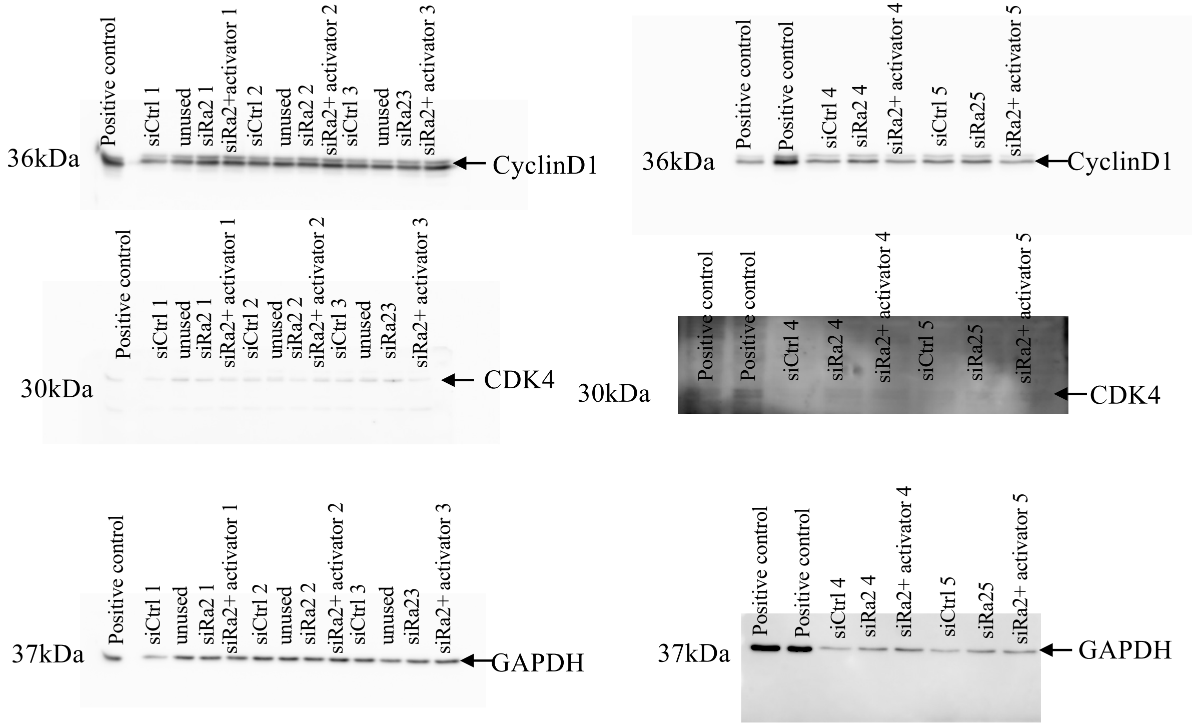

siCtrl indicates Control group.
siRa2 indicates knockdown group.
siRa2+agonist indicates knockdown cells treated with activator.

Figure S2. Total cell numbers quantified in the same microscopic fields as BrdU analysis.

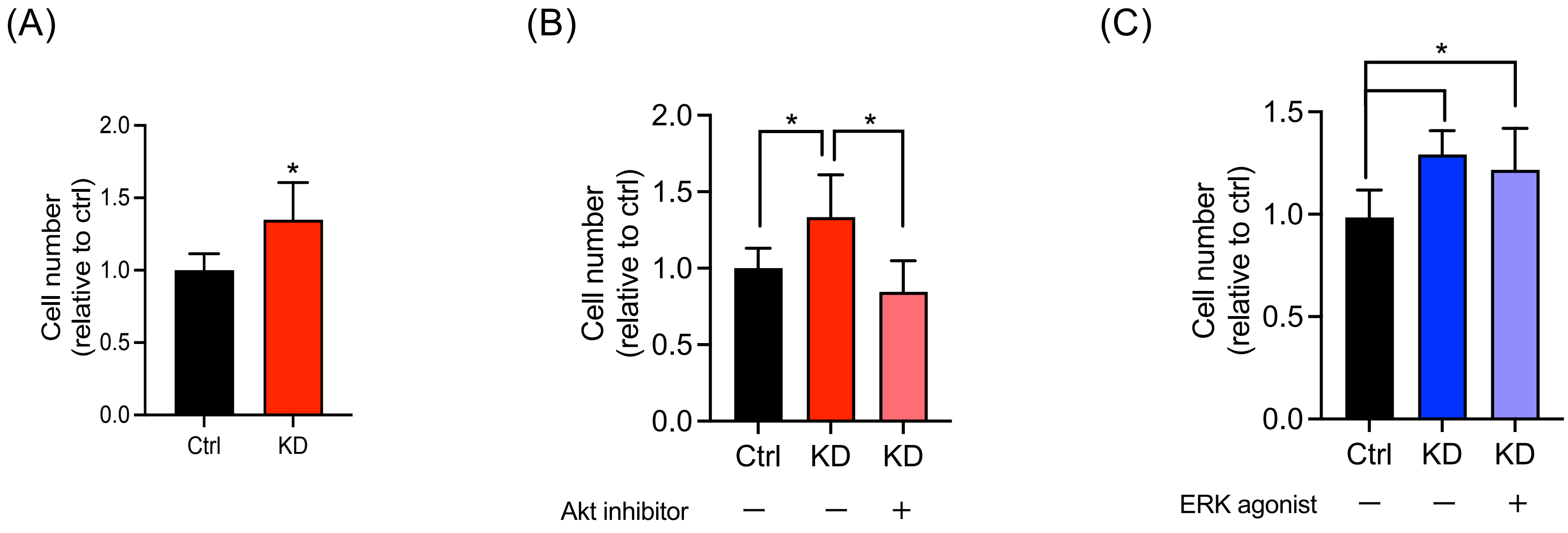

(A) Quantification of total cell numbers in Ctrl and KD groups. Data correspond to the fields analyzed in Figure 2B. $N = 6$ per group.
(B) Total cell numbers in Ctrl, KD, and KD + Akt inhibitor groups. Data correspond to the fields analyzed in Figure 3B. $N = 6$ per group.
(C) Total cell numbers in Ctrl, KD, and KD + ERK activator groups. Data correspond to the fields analyzed in Figure 4B. $N = 6$ per group.
Total cell numbers were determined by counting DAPI-positive nuclei in the identical microscopic fields used for BrdU incorporation. Data are presented as mean ± SD. Statistical significance was determined by an unpaired Student's *t*-test or one-way ANOVA followed by Tukey–Kramer post hoc test. * $p < 0.05$.

Table S1. Primer sequences used in qRT-PCR

	Primer sequence	
Target	Forward (5’-3’)	Reverse (5’-3’)
GAPDH	TGTGTCCGTCGTGGATCTGA	TTGCTGTTGAAGTCGCAGGAG
Myogenin	CAGTGAATGCAACTCCCACAG	TGGACGTAAGGGAGTGCAGA
Myomaker	ATCGCTACCAAGAGGCGTT	CACAGCACAGACAAACCAGG
Myomerger	CAGGAGGGCAAGAAGTTCAG	ATGTCTTGGGAGCTCAGTCG
MyHC4	GAGTTCATTGACTTCGGGATGG	TGCTGCTCATACAGCTTGTTCTTG
Cyclin D1	TGACTGCCGAGAAGTTGTGC	CTCATCCGCCTCTGGCATT
CDK4	AAGGTCACCCTAGTGTTTGAGC	CCGCTTAGAAACTGACGCATTAG
